# Perception of Elasticity in the Kinetic Illusory Object with Phase Differences in Inducer Motion

**DOI:** 10.1371/journal.pone.0078621

**Published:** 2013-10-25

**Authors:** Tomohiro Masuda, Kazuki Sato, Takuma Murakoshi, Ken Utsumi, Atsushi Kimura, Nobu Shirai, So Kanazawa, Masami K. Yamaguchi, Yuji Wada

**Affiliations:** 1 Food Function Division, National Food Research Institute, National Agriculture and Food Research Organization, Ibaraki, Japan; 2 Department of Psychology, Chuo University, Tokyo, Japan; 3 Behavioral and Cognitive Neuroscience Unit, RIKEN BSI-TOYOTA Collaboration Center, Saitama, Japan; 4 Department of Information Environment, Tokyo Denki University, Chiba, Japan; 5 Department of Psychology, Niigata University, Niigata, Japan; 6 Department of Psychology, Japan Women's University, Kanagawa, Japan; University of Regensburg, Germany

## Abstract

**Background:**

It is known that subjective contours are perceived even when a figure involves motion. However, whether this includes the perception of rigidity or deformation of an illusory surface remains unknown. In particular, since most visual stimuli used in previous studies were generated in order to induce illusory rigid objects, the potential perception of material properties such as rigidity or elasticity in these illusory surfaces has not been examined. Here, we elucidate whether the magnitude of phase difference in oscillation influences the visual impressions of an object's elasticity (Experiment 1) and identify whether such elasticity perceptions are accompanied by the shape of the subjective contours, which can be assumed to be strongly correlated with the perception of rigidity (Experiment 2).

**Methodology/Principal Findings:**

In Experiment 1, the phase differences in the oscillating motion of inducers were controlled to investigate whether they influenced the visual impression of an illusory object's elasticity. The results demonstrated that the impression of the elasticity of an illusory surface with subjective contours was systematically flipped with the degree of phase difference. In Experiment 2, we examined whether the subjective contours of a perceived object appeared linear or curved using multi-dimensional scaling analysis. The results indicated that the contours of a moving illusory object were perceived as more curved than linear in all phase-difference conditions.

**Conclusions/Significance:**

These findings suggest that the phase difference in an object's motion is a significant factor in the material perception of motion-related elasticity.

## Introduction

**Figure 1:**
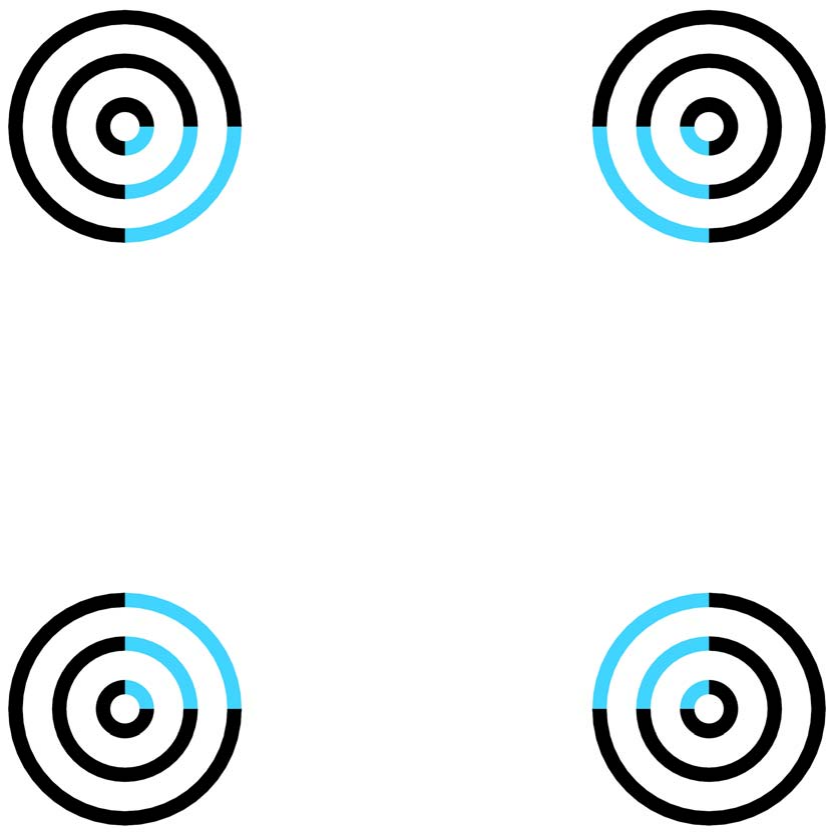
Example of neon color spreading.

Subjective contours (SC) are perceived edges between surfaces without actual luminance or color differences across their edges [1], [2]. For example, observers see an illusory triangle when three black disks with wedge-shaped cutouts are arranged to form the three corners of a triangle. Additionally, the illusory surface with SC is perceived with a tinted color when colored segments, rather than blank spaces, are inserted into the cutout areas of inducers [3], [4], a phenomenon known as neon color spreading (NCS) (Figure 1). This effect is a known illusory phenomenon in which observers perceive tinted color in achromatic space among colored inducers, as in the watercolor effect (e.g., [5]–[7]).

Three-dimensional shapes can also be perceived as illusory objects with SC and the NCS effect [8], [9]. It is known that SC are perceived even if the figures involve motion. When the inducers of a subjective contour are deformed continuously, the moving subjective contour can be perceived even where a motionless subjective contour cannot (Kinetic SC) [10]. Bressan and Vallortigara [11] reported that observers perceived a moving illusory bar in a rotating pattern composed of concentric circles that were interrupted as if by a bar extending from the center to the periphery. These studies suggest that SC can be clearly perceived with motion and that illusory phenomena include the brightness, color and three-dimensional shape of the illusory object (see Bressan, Mingolla, Spillmann & Watanabe [12] for a comprehensive review). In addition, we reported that even 7- to 8-month-old infants can discriminate between kinetic NCS and non-NCS figures that include phase differences caused by inducer motions [13]. Furthermore, Single-cell activities in primates [14] and neuroimaging in humans [15] have suggested the importance of the early stage of visual processing in the visual cortex (V2, V5, and, prominently, V1), showing that both moving and static SC activate low-order visual areas [16].

In studies of motion perception, a three-dimensional structure perceived from motion is described in relation to its rigidity [17], [18]; however, such perception of an illusory surface with SC remains unexplored. Whether material properties such as rigidity or elasticity can be perceived in illusory surfaces has not been examined since almost all visual stimuli used in previous studies have been generated to induce rigid illusory objects. Here, we elucidate whether a kinetic illusory object with SC might involve some motion percept caused by differences in material property such as rigidity or elasticity. “Rigidity” indicates a property of materials that maintain their original shape when they are subjected to external forces, whereas “elasticity” indicates a property of materials that return to their original shape after force is applied to the object. Thus, a rigid object is undeformable, and, in contrast, an elastic object changes in shape (non-rigid) and has varied deformation behavior after a force is applied to it, depending on its degree of elasticity.

The motions of non-rigid objects are ubiquitous not only in SC, but also in nature. However, research on non-rigid perception has been limited [19], although some studies have reported that humans perceive non-rigid objects as having various rigidities and elasticities [20], [21]. For example, Johansson [20] demonstrated that observers perceived the motion of a square continuously changing to a rectangle in the 2D plane as rigidly rotating in depth or non-rigidly folding or bending in depth. Jansson and Johansson [21] also found that observers tended to perceive rotation, bending, and stretching when they were shown the transformation of a quadrangle with various combinations of changes in length and direction of the four sides. Recently, it was reported that the perpendicular square-wave grating pattern was perceived as non-rigid horizontal compression and expansion when observers viewed the grating pattern with back and forth head movement along the projection line (Accordion Grating illusion) [22], [23]. Perceived non-rigid distortion in the Accordion Grating illusion cannot be explained by the traditional 2D aperture problem and 3D visual space plays a crucial role in its mathematical analysis [23].

In nature, the actual motion or deformation of an object varies according to its material properties. For instance, the peak height of a bouncing object varies depending on the object's elasticity. The amount and type of deformation also differs depending on an object's hardness when a force is exerted on it.

Hence, we can assume that visual motion might provide rich information for material perception. Indeed, several reports have indicated that observers perceive some material properties of objects. For instance, observers can perceive a moving object's elasticity based on velocity changes around its collision with the floor or from the amplitude of its bounce [24]. Moreover, Masuda, Kimura, Goto and Wada [25] reported that the visual hardness perception of a penetrated object was influenced by the penetrating object's velocity changes and average velocity during penetration. These studies have indicated that velocity change is a determinant for the perception of material property from motion. Thus, we can assume that the potential link between the determining factor of material perception from visual motion and the mechanical behavior varies with the type of material. For example, Masuda et al. [25] suggested that observers judge an object's hardness based on visual motion similar to a physical penetration of an object with a hard surface. They showed that the visual hardness perception in a penetration event may also be described as a motion based on Newton's laws of motion because the penetrating object's velocity varies depending on the exerted force related to the material property of the penetrated object. Given that changes in an object's motion can be caused by the mechanical behavior related to elasticity, phase differences of oscillation between the parts of a non-rigid object should also contribute to apparent elasticity. For example, in the mechanical behavior of materials, temporal phase differences between the stress point and deformation point vary depending on viscoelastic properties, which have combined elasticity and viscosity, when the object is stressed. This difference reaches 90 deg. as the elasticity of the object decreases (liquid-like material), and declines to 0 deg. as the elasticity of the object increases (solid-like material) in material mechanics [26].

Here, we have attempted to reveal the effect of oscillation phase differences with SC on the visual impressions of an object's elasticity. In Experiment 1, we investigated whether the material perception of SC varied with phase differences between oscillations in induced figures. Using an illusory figure with kinetic SC, rather than actual contours, allowed us to manipulate the degree of phase difference systematically without actual contour deformation. Additionally, we used SC with the NCS effect to make judging a subjective figure easier for participants because a moving subjective figure is perceived more vividly in a NCS figure than in a Kanizsa-type figure [27].

Next, Experiment 2 was designed to examine whether the perceived elasticity of a moving object depends on its shape or on the phase difference included in the perceived object. Wallach and O’Connell [28] demonstrated that observers watching the silhouette of a rotating linear rigid wire with clear vertices projected onto a screen tended to perceive a rigid rotating 3-D structure (kinetic depth effect), whereas they tended to perceive a distorted object when the wire was curved without salient vertices. Thus, the linearity of a moving object's shape is considered to be associated with the object's rigidity or elasticity. Given the influences of the object's shape linearity, it is possible that the differences in linearity of a subjective object's contours influence the material perception in kinetic SC similar to the kinetic depth effect. If the contour shape is the only cue for rigidity or elasticity, observers should perceive linear SC as rigid motion whereas they should perceive curved SC as non-rigid (elastic) motion.

On the other hand, Kellman and Shipley [29] proposed the theory of *contour relatability*: that we can perceive SC with curved lines when the edges of inducers are connected by an interpolated contour that is smooth and bends between the edges of inducers. Based on this theory, we can expect that SC with motion in the current study will be perceived as curved shapes under any phase difference condition or with any perceived elasticity.

We conducted Experiment 2 in order to clarify the perceived shape of SC. We used multidimensional scaling (MDS) analysis for similarity ratings between pairs of kinetic SC and actual contours drawn with straight or curved lines. The MDS allows us to investigate how observers perceived the shape of kinetic SC in each phase difference condition. If SC were always perceived as curved shapes, as the contour reliability theory predicts, results would indicate that not only the shape of the SC, but also the combination of phase difference of motion in an object and the shape of the SC are cues for elasticity.

Our purpose was to confirm whether the magnitude of phase difference in oscillation influences the visual impressions of an illusory object's elasticity (Experiment 1) and to identify whether such elasticity perceptions are accompanied by the shape of the SC, which are assumed to be strongly correlated with the perception of rigidity (Experiment 2).

## Experiment 1

### Materials and Methods

#### Ethics statement

This research followed the tenets of the Declaration of Helsinki. Written informed consent was obtained after a complete explanation of the study. The study was approved by the institutional ethics committee of the National Food Research Institute.

#### Participants

Ten healthy adults (six women, four men) aged 21–45 years participated in the experiment. They all had normal color vision and normal or corrected-to-normal visual acuity.

#### Apparatus

Visual stimuli were controlled using a personal computer (Dell, Precision 390) and displayed at the center (600×600 pixels; 9.26×9.26 deg. in visual angle) of a 22-in. CRT monitor (resolution: 1024×768 pixels; diagonal visual angle: 19.72 deg.; Iiyama, HM204DA; 120 Hz refresh rate).

#### Visual Stimuli

The stimuli consisted of four sets of three concentric circles (diameter: outer circle 1.98 deg., middle circle 1.23 deg., and inner circle 0.49 deg. in visual angle) presented on a white background. The line width in each circle was 0.12 deg. Each set of circles was placed in one corner of a square (distance between centers: 4.94 deg. in visual angle). The constructional lines of a quarter segment of each set of circles were painted blue to cause the perception of a faint bluish color within a square region (Figure 1).

**Figure 2:**
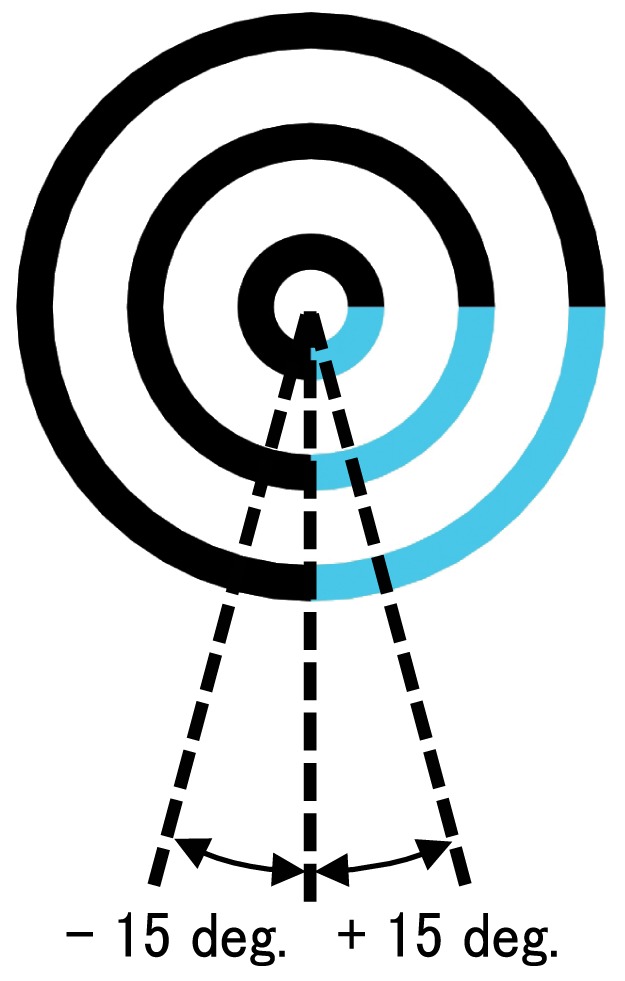
Schematic display of the pendulum motion used in this experiment.

The vertical boundaries of the painted regions oscillated around the center of the concentric circles. The oscillation angle shifted from −15 deg. to +15 deg. (Figure 2). Each pattern of oscillating motion was presented for 3.0 s, after the first frame in motion was presented for 0.5 s.

**Figure 3:**
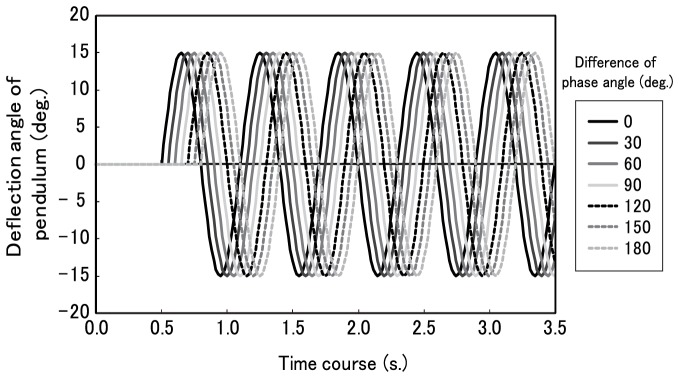
Mode of phase differences in Experiment 1 (low velocity pendulum condition: 1.72 deg. per s. in visual angle). The horizontal axis indicates the time course (s) of the oscillating motion, and the vertical axis indicates the oscillation angle (deg.).

We manipulated the phase difference between pendular motions in the upside and downside vertical boundary oscillations. Phase differences were implemented in seven steps (0, 30, 60, 90, 120, 150, and 180 deg.) by delaying the beginning of the pendular motion (Figure 3). Pendular motions in the right and left boundaries were parallel. In order to manipulate the velocity of the pendulum, we adjusted the number of cycles per second to high (2.50 cycles per s.) or low (1.67 cycles per s.). The velocity of the pendulum changed periodically resulting from a sinusoidal pendulum oscillation. The average velocity was either 2.59 deg. per s. in visual angle (high-velocity pendulum condition) or 1.72 deg. per s. in visual angle (low-velocity pendulum condition). Experiment 1 stimuli are shown in Videos S1–S4 (supporting information available on-line; Videos S1-S4: 0-, 30-, 90-, 180-deg. phase difference under the low-velocity pendulum condition, in that order).

To cancel out asymmetric stimuli orientation, we made horizontally and/or vertically flipped patterns of all stimuli, an equal number of which were presented in the experiment, generating a total of 56 patterns (7 phase difference conditions×2 velocity of pendulum conditions×2 horizontal positions×2 vertical positions). The horizontal flip reversed the initial horizontal direction of the pendular motion starting to the right or to the left. The vertical flip reversed the initial vertical direction of the pendular motion starting from the upside or the downside. The phase difference is caused by differences in the motions of the upside and downside inducers, thus the vertical flip did not result in a change in the patterns without a phase difference. All stimuli were presented at 60 frames/s.

#### Procedure

The experiment was conducted in a dark room. After 10 min of dark adaptation, participants were instructed in the experimental task and procedures. A participant's head was fixed on a chin rest, and the viewing distance from the observer to the monitor was approximately 143 cm. All 56 visual patterns were presented in random order, different for each participant, with five repetitions. In total, each participant underwent 280 trials.

At the beginning of the experiment, a static figure with the same form as the figures used during the experimental session was presented in order to demonstrate the SC and NCS effect to each participant. All participants reported that they perceived the SC and illusory surface.

After a participant observed a motion stimulus, they were asked which of the following best described their perception of the visual stimulus: “a swinging, rigid convex or concave plane in depth” (concave/convex), “a swinging, rigid S-shaped plane in depth” (S-shaped), “bending”, “waving”, or “an illusory object was not seen” (unseen). Observers were instructed in these five motion categories as follows. Responses of “a swinging, rigid convex or concave plane in depth” (concave/convex) or “a swinging, rigid S-shaped plane in depth” (S-shaped) indicate that a swinging object is perceived in depth with no deformation in the illusory figure. A response of “bending” indicates that the motion of a high-elasticity material, such as a tree branch or a thin plastic ruler, is perceived in the illusory figure. A response of “waving” indicates that the motion of a low-elasticity material, such as a wind-whipped flag, is perceived in the illusory figure. A response of “an illusory object was not seen” indicates that no illusory contours are perceived in the visual pattern. These responses were based on free descriptions generated by three participants who observed the visual stimuli in a pilot study.

#### Data analysis

For each participant, we calculated the rate of each response across all conditions. No differences for initial direction of motion were found using any dependent measure, thus, for the sake of simplicity, direction of motion has been excluded from the reported analyses. The rates for each impression were analyzed with a two-way analysis of variance (ANOVA) with within-subject factors between phase difference and pendulum velocity. Effect sizes (partial eta squared: *η_p_^2^*) were also calculated. When significant effects were detected, *post*-*hoc* multiple comparisons of means were performed using Tukey's test. *P*<.05 was considered statistically significant.

## Results

**Figure 4:**
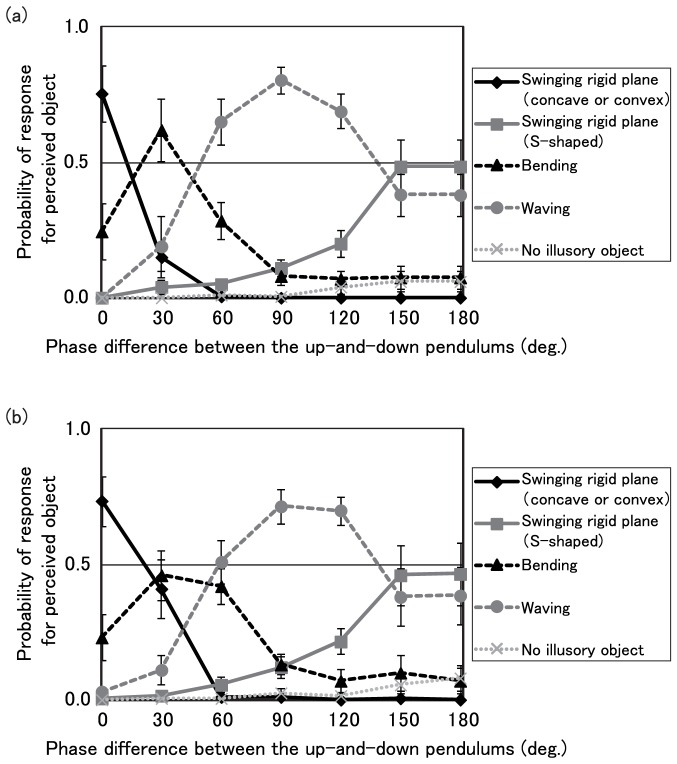
Mean ratings categorized as motion impressions in each phase difference condition under (a) low and (b) high oscillation velocity conditions. The horizontal axis indicates the phase difference (deg.), and the vertical axis indicates the mean probability of rating for perceived object. Error bars indicate standard error (*N*  = 10).

Figures 4a and 4b show the mean ratings of responses for the five categories under each pendulum velocity condition.

### 

#### “Swinging, rigid convex or concave plane in depth” ratings

A 7×2 (phase difference×pendulum velocity) within-subjects ANOVA was performed on the “convex/concave” ratings. We found main effects of phase difference (*F*(6, 54) = 36.62, *p<*.01, *η_p_^2^* = .80), pendulum velocity (*F*(1, 9) = 10.60, *p<*.01, *η_p_^2^* = .16), and interaction between factors (*F*(6, 54) = 10.29, *p<*.01, *η_p_^2^* = .53). Post-hoc analysis revealed that the simple main effects of phase difference were significant for the high- and low-velocity pendulum conditions, and the simple main effect of pendulum velocity was significant for the phase difference of 30 deg. Tukey's post-hoc tests indicated that the ratings of “convex/concave” for the phase difference of 0 deg. were higher than those for the other phase difference conditions in the low-velocity pendulum condition (*p*<.05), and that ratings for the phase differences of 0 and 30 deg. were higher than those for the other phase difference conditions in the high-velocity pendulum condition (*p*<.05). In the high-velocity pendulum condition, the ratings for the phase difference of 0 deg. were also higher than those for the 30-deg. condition.

These results suggest that observers tended to perceive the swinging of a rigid object under conditions where the phase difference was close to 0 deg.

#### “Swinging, rigid S-shaped plane in depth” ratings

A 7×2 (phase difference×pendulum velocity) within-subjects ANOVA was performed on the “S-shaped” ratings. We found a main effect of phase difference (*F*(6, 54) = 13.40, *p<*.01, *η_p_^2^* = .60). Tukey's post-hoc tests indicated that ratings of “S-shaped” for phase differences of 150 and 180 deg. were higher than those for the other phase difference conditions (*p<*.05).

These results suggest that observers tended to perceive the swinging of a rigid object under conditions where the phase difference was close to 180 deg.

#### “Bending” ratings

An ANOVA was performed on the “bending” ratings. We found main effects of phase difference (*F*(6, 54) = 7.08, *p<*.01, *η_p_^2^* = .44), and interaction between factors (*F*(6, 54) = 3.12, *p<*.05, *η_p_^2^* = .26). Post-hoc analysis revealed that the simple main effects of phase difference were significant for the high and low-velocity pendulum conditions, and the simple main effects of pendulum velocity were significant for the phase differences of 30 and 60 deg. Tukey's post-hoc tests indicated that ratings of “bending” for the phase difference of 30 deg. were higher than those for 0, 90, 120, 150, and 180 deg. in the low-velocity pendulum condition (*p*<.05), and that ratings for the phase difference of 30 deg. were higher than those for 120, 150, and 180 deg. in the high-velocity pendulum conditions (*p*<.05).

These results suggest that observers tended to perceive a bending motion under conditions where the phase difference was close to 30 deg.

#### “Waving” ratings

An ANOVA was performed on the “waving” ratings. We found a main effect of phase difference (*F*(6, 54) = 16.87, *p<*.01, *η_p_^2^* = .65). Tukey's post-hoc tests indicated that ratings of “waving” for the phase difference of 90 deg. were higher than those for 0, 30, 150, and 180 deg. (*p*<.05), that ratings for the 60-deg. difference condition were higher than those for 0 and 30 deg. (*p*<.05), and that ratings for the 120-deg. difference condition were higher than those for 0 and 30 deg. (*p*<.05).

These results suggest that observers tended to perceive a bending motion under conditions where the phase difference was close to 90 deg.

#### “Illusory object was not seen” ratings

An ANOVA was performed on the “unseen” ratings. We found a main effect of phase difference (*F*(6, 54) = 2.57, *p<*.05, *η_p_^2^* = .22). Tukey's post-hoc tests indicated no significant differences between conditions (*p*>.05). Additionally, a student t-test (FDR*-*corrected for multiple comparisons; *q* = 0.05) showed that the response rating did not deviate significantly from zero in each experimental condition (*p*<FDR 0.05).

These results suggest that most observers perceived the illusory figure under all conditions.

Experiment 1 revealed that the rate of perception of elastic objects changed as a function of temporal difference when the temporal differences between inducers' oscillations were included in an illusory object. The perception of rigid motion was maximized at the phase difference of 0 deg., that of bending at 30 deg., and that of waving at 90 deg. In other words, the results of Experiment 1 indicate that the perceived material impressions related to the degree of an object's elasticity could be systematically flipped by the phase difference in visual motion.

## Discussion

The results of Experiment 1 demonstrate that illusory surfaces with SC were perceived under all phase difference conditions, and the perceived elasticity of the illusory surfaces changed with the phase differences within the object.

The swinging rigid plane impression, which includes the illusory surface in depth, was dominant when the phase difference was near 0 deg. This is consistent with previous studies [30]–[32]. In contrast, waving motion impressions increased as the phase difference shifted closer to 90 deg. Moreover, bending motion impressions were dominant at 30 deg. This implies that material perceptions based on motion might be determined by the phase differences between inducer motion: in nature, actual rigid swinging, bending, and waving motions occur with undeformable rigid, high-elasticity, and low-elasticity materials, respectively. Therefore, our results indicate that the visual perception of material based on motion might be achieved using naturally occurring motion. Which is to say that degrees of phase difference systematically vary elasticity perceptions.

Additionally, Experiment 1 results show the effect of pendulum velocity under the 30- and 60-deg. phase difference conditions. These differences might be attributed to the judgment of temporal differences in the inducers. In the high-velocity conditions, the temporal differences in the inducers were 1/30 s. in the 30-deg. phase difference condition. Thus, the detection of phase differences should be difficult for observers under high-velocity pendulum conditions. Such difficulties at 30 deg. might enhance the swinging impression, which tended not to be perceived in other phase difference conditions. Similarly, in the high-velocity condition, a 60-deg. phase difference might be necessary for the appearance of a phase difference similar to that of the 30-deg. phase difference condition in the low-velocity pendulum condition.

Rates of waving and s-shaped rotating impressions were the same in the vicinity of the 180-deg. phase difference. At 180 deg., a long time lapse after the start of one inducer's motion and before the start of another's was required to create the large phase differences. Deformed SC, such as a waving impression, might be perceived in this partial motion condition. While there may be another determinant for distinguishing between these impressions, these results have at least revealed that only two types of motion tend to be perceived in the 180-deg. phase difference condition.

For material mechanics, the inference of material from phase difference was indicated in the 0- to 90-deg. range [26]. The pendular motions in the angle of phase differences between 90 and 180 deg. execute reverse direction movements to those in the angle of phase differences between 0 and 90 deg. (opposite phase). Thus, the difference of temporal change reaches its peak at 90 deg., after which the differences of temporal change decrease as the inverse of an angle in phase degrees.

Our results imply that observers judge illusory surface material based on visual motion similar to the physical motion of an oscillation caused by certain types of material. Thus, observers perceived highly rigid (or elastic) surfaces (swinging-rigid motion) in the 0- and 180-deg. phase difference conditions and less rigid (or elastic) surfaces (waving motion) around the 90-deg. phase difference conditions.

Experiment 1 showed that the material perception of an illusory surface varied with phase differences between inducers. It is known that the type of shape of an object, such as the linearity of a contour with vertices, is related to the perception of an object's rigidity [28]; however, it is still unclear whether the shapes of SC are perceived as rectilinear or sweeping forms. To clarify this issue, we used MDS analysis for similarity ratings between pairs of kinetic SC and actual contours drawn with straight or curved lines.

## Experiment 2

### Materials and Methods

#### Ethics statement

This research followed the tenets of the Declaration of Helsinki. Written informed consent was obtained after a complete explanation of the study. The study was approved by the institutional ethics committee of the National Food Research Institute.

#### Participants

Ten healthy adults (4 women, 6 men) aged 31–49 years participated in the experiment. They all had normal or corrected-to-normal visual acuity. Three of the ten also participated in Experiment 1.

#### Apparatus

Visual stimuli were controlled using a personal computer (Dell, Precision 390) and displayed on a 22-in. CRT monitor (resolution: 1024×768 pixels; diagonal visual angle: 22.05 deg.; Iiyama, HM204DA; 120 Hz refresh rate).

#### Visual stimuli

**Figure 5:**
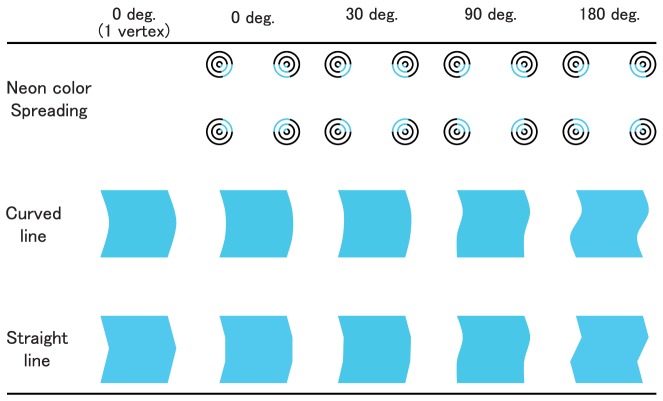
The types of figure presented in Experiment 2.

Stimuli with the same pendulum motions as those used in Experiment 1 were created with actual angular or smoothly curved contours. Curved vertical contours, horizontal contours, and other shapes were depicted as smooth splines, straight lines, and straight lines, respectively (Figure 5). The two vertices for drawing each type of actual line were placed in the same position: the distance between the center of an inducer and a vertex and the distance between vertices at the beginning of pendular motion were the same. The vertices maintained the same distance from the end of inducers during pendular motion. We generated visual stimuli under four phase difference conditions (0, 30, 90, and 180 deg.) for each of three types of contours (neon-color shape (NCS), curved line shape (CL), and straight line (SL)). Additionally, a single vertex was used in the 0-deg. condition. The duration of oscillation was identical to that of the low-velocity condition in Experiment 1 (1.67 cycles per s.).

Thus, a total of 14 visual patterns were generated (3 shape conditions× 4 phase difference conditions + 2 single-vertex conditions). The size of each pattern was 9.03×9.03 deg. in visual angle (468×468 pixels).

#### Procedure

The experimental setup was similar to that of Experiment 1 except for viewing distance, which was approximately 114 cm from observer to monitor. For each trial, two visual stimuli, arranged side-by-side on a gray background 1.70 deg. apart in visual angle (88 pixels), were presented on the monitor. Each participant underwent 182 trials consisting of pairs of each of the 14 visual patterns (91 pairs×2 alignments, so that each pattern was observed in both the left and the right positions) in random order. Participants could observe the stimuli as often as they needed to rate the similarity between the shapes. For similarity ratings, participants checked any point along a visual analog scale ranging from "very similar" to "very dissimilar". Using a linear scale, "very similar" was assigned a value of 0 and "very dissimilar" was assigned a value of 10.

#### Data analysis

To characterize the apparent shape of the SC, we analyzed the similarity ratings for each pair of visual stimuli, converted them to dissimilarities, and averaged them across participants. The dissimilarity ratings were subjected to MDS analysis using an alternating least squares scaling method (ALSCAL) and a standard Euclidean model. In addition, to investigate similarities among the apparent shapes of subjective straight and curved contours, we calculated the Euclidian distances between line-plot types under the same phase difference conditions.

## Results

**Figure 6:**
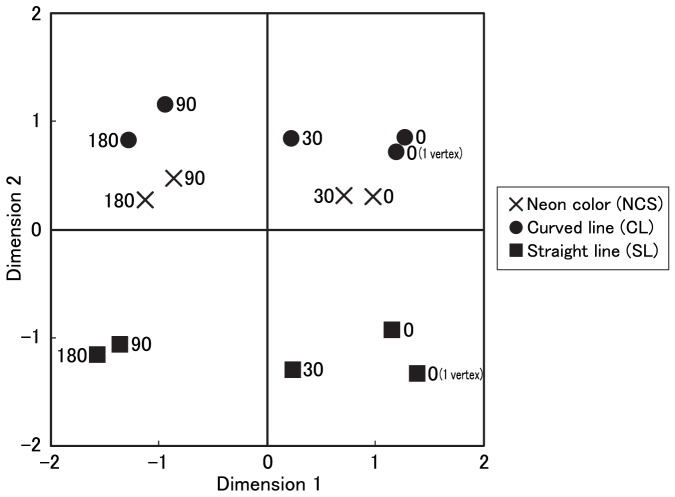
Representations of stimuli in two-dimensional space. The crosses, circles, and squares represent figure types from neon color spreading, curved line, and straight line conditions, respectively. The numbers 0, 30, 90, 180 designate phase differences. The dimension 1 is interpreted as the “angle of phase differences”. The dimension 2 is interpreted as “shape of contours”.

The 2-dimensional solution shown in Figure 6 was obtained. In the solution of MDS for two dimensions, the Kruskal's Stress measure [33] was.15, and *r^2^* was .87. Each symbol indicates the type of shape, and the pairwise distances between symbols indicate corresponding pairwise perceptual dissimilarities between the shapes of visual stimuli.

The first dimension was interpreted as the “angle of phase differences” dimension. Stimuli for each of three types of contours were located in order of degree of phase difference on this dimension. The second dimension was interpreted as the “shape of contours” dimension. On this dimension, NCS was inserted between straight and curved lines. In addition, the distance between NCS and CL was shorter than that between NCS and SL.

**Figure 7:**
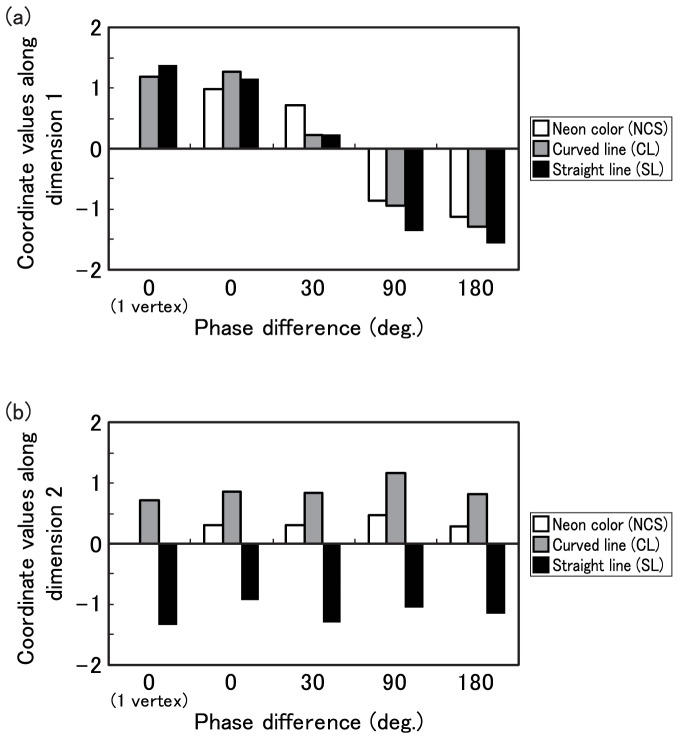
Coordinate values along dimension 1 and 2. The horizontal axis indicates the phase difference (deg.), and the vertical axis indicates the coordinate value for (a) dimension 1 and (b) dimension 2 for each shape condition.

Figures 7a and 7b show coordinate values for the first and second dimensions for each visual pattern. These figures confirm that the first and second dimensions robustly correspond to phase differences and shapes of contours. In particular, differences between NCS and CL are constantly smaller than those between NCS and SL, whereas differences between CL and SL are greatest.

**Figure 8:**
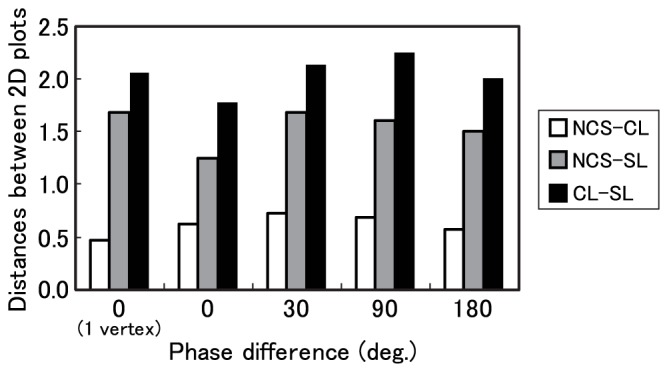
Distance between the plot of neon color spreading (NCS), curved line (CL), and straight line (SL) under the same phase difference conditions in MDS space, as a function of phase differences. The horizontal axis indicates the phase difference (deg.), and the vertical axis indicates the Euclidian distance between plots for each pattern.

Figure 8 shows the distance between NCS, CL and SL. We found that the distance between NCS and CL was the smallest among these distances for all phase difference conditions.

## Discussion

We examined whether phase difference influences the linearity of SC in Experiment 2. As a result of MDS, a 2-dimensional solution was obtained. The obtained dimensions are represented as “phase difference” and “shape of contours”. The distances in each dimension and the obtained 2D space between each object's location correspond to the degree of dissimilarity between pairs of objects. The distance between NCS and curved contours in 2D space and in second dimension was consistently shorter than other distances.

These results indicate that the apparent shape of contours in NCS is more similar to curved than to straight contours. The relationship between contours was consistent across all phase difference conditions. These results imply that the shapes of SC were perceived as smooth curved edges in kinetic conditions, even when there were phase differences between inducer oscillations. In other words, SC did not tend to appear as linear contours in any phase difference condition, whereas the rate of rigid motion was very high under the 0-deg. condition in Experiment 1. Thus, the theory that the perceived elasticity of kinetic illusory surfaces is influenced by both the shapes of perceived contours and the phase differences of an object is supported.

## Conclusion

We found that the kinetic subjective contours were perceived even if the stimuli involved phase differences accompanied with various elasticity perceptions of illusory surfaces. Furthermore, the material impressions related to elasticity were systematically flipped by the phase difference in visual motion.

The results of Experiment 1 reveal that variations in phase difference can have a large effect on how an illusory surface is perceived. The rate of rigid motion is maximized for the phase difference of 0 deg., the rate of bending motion is maximized for the phase differences of 30 deg., and the rate of waving motion is maximized for the phase differences of 90 deg.

Thus, rigid objects tended to be perceived under conditions in which the direction of each inducer's oscillation changed simultaneously, such as the 0 and 180 deg. phase difference conditions. On the other hand, when the temporal differences between inducers' oscillations were included in an illusory object, several types of elastic objects tended to be perceived as a function of temporal difference.

Results of Experiment 2 show that the shapes of kinetic subjective contours in all phase difference conditions are perceived as vertical, smooth, curved contours.

Taken together, the results of our experiments indicate that motion impression responses vary with phase differences between inducers, but preserve smooth curved contours, implying that perceived material elasticity based on motion might be partially determined by the phase difference included in an object's deformation. Actual rigid swinging, bending, and waving motions occur with undeformable rigid, high-elasticity, and low-elasticity materials, respectively, in nature. Therefore, the current results imply that the visual perception of material based on motion is achievable using naturally occurring motion cues of various materials. Some studies have also reported changes in the shapes of illusory figures with motion (e.g., [34]). They argued that the mechanism of contour synthesis in illusory figures instantiates a process of ideal rational inference. Our findings suggest that “rational” material properties also associated with the surface of an object might be included in the mechanism of contour synthesis.

Recently, we reported that 7- to 8-month-old infants could discriminate between NCS and non-NCS with a 90-deg. phase difference while 3- to 6-month-olds could not [13]. Current results confirm that visual stimuli with a phase-differing movement of inducers, in which 7- to 8-month-old infants potentially perceive illusory contours, elicit the perception of non-rigid dynamic subjective contours in adults. This phenomenon may be a cue for human development of visual material and rigidity perception, and should be explored further in future research.

It remains unclear which, the processing of shape or of material perception, is earlier. One possibility is that curved SC induce the impression of a non-rigid material. However, our results indicate that elasticity impressions were flipped as the degree of phase difference increased, while the perceived SC remained similar to curved contours under all phase differences. Thus, it is clear that phase difference included in an object's motion is one determinant of material perception based on elasticity.

In dynamic NCS, Cicerone and Hoffman [35] found that the perceptual attributes of motion, color, form, and depth can be grouped and regrouped by our visual systems into different combinations. They provided perceptual evidence for selective activation of multiple visual pathways. They proposed a relationship between these effects and the physiological results from the middle temporal visual area known as V5 (MT). Furthermore, the V5 (MT) associated with actual motion perception is also related to illusory motion without actual visual motion by the Enigma illusion [36]. Sasaki and Watanabe [37] also observed activity for SC in the ventral pathway, which includes multiple visual areas (V1, V2, V3/VP, and V4v), and suggested that SC and color spreading were processed separately. We found that kinetic SC with neon color were accompanied by various elasticities; the visual phenomena that we found can be assumed to relate not only to the ventral pathway, but also to the dorsal pathway because they include both of SC and motion. Although the ventral cortex around the fusiform gyrus is related to categorization of materials in humans [38], the neural pathway for inferring an object's material based on motion remains unclear. Our findings might be a strong tool for investigating the mechanism of kinetic material perception.

In Experiment 1, we used a five-choice forced-choice task to explore the perceived material, and calculated the rate of each response in order to clarify the broad effect of phase difference on material perception. The relationship between the gradual change of each perceived material property (rigidity, elasticity, and so on) and the gradual change of phase differences has to be elucidate using other psychophysical techniques, such as the direct rating of magnitude for some material property used in another area of investigation such as sensory evaluation, material mechanics, and so on.

In conclusion, the current results provide evidence of the relationship between material impressions related to the degree of an object's elasticity in kinetic SC with a smooth curved shape and the degree of the phase difference in inducers' pendulum motion. These findings are an important step towards understanding material perception from visual motion.

## Supporting Information

Video S1 **0-deg. phase difference under low-velocity pendulum condition.**
(AVI)

Video S2 **30-deg. phase difference under low-velocity pendulum condition.**
(AVI)

Video S3 **90-deg. phase difference under low-velocity pendulum condition.**
(AVI)

Video S4 **180-deg. phase difference under low-velocity pendulum condition.**
(AVI)
